# Complement biomarkers reflect the pathological status of neuromyelitis optica spectrum disorders

**DOI:** 10.3389/fimmu.2023.1090548

**Published:** 2023-03-03

**Authors:** Katsuichi Miyamoto, Mai Minamino, Motoi Kuwahara, Hiroshi Tsujimoto, Katsuki Ohtani, Nobutaka Wakamiya, Kei-ichi Katayama, Norimitsu Inoue, Hidefumi Ito

**Affiliations:** ^1^ Department of Neurology, Wakayama Medical University, Wakayama, Japan; ^2^ Department of Neurology, Kindai University School of Medicine, Osaka-sayama, Japan; ^3^ Department of Molecular Genetics, Wakayama Medical University, Wakayama, Japan; ^4^ Department of Clinical Nutrition, Rakuno Gakuen University, Ebetsu, Japan; ^5^ Department of Medicine and Physiology, Rakuno Gakuen University, Ebetsu, Japan

**Keywords:** neuromyelitis optica spectrum disorders, Guillain-Barré syndrome, complement, alternative pathway, sC5b-9, CFH, Ba

## Abstract

Complement is involved in the pathogenesis of neuroimmune disease, but the detailed pathological roles of the complement pathway remain incompletely understood. Recently, eculizumab, a humanized anti-C5 monoclonal antibody, has been clinically applied against neuroimmune diseases such as myasthenia gravis and neuromyelitis optica spectrum disorders (NMOSD). Clinical application of eculizumab is also being investigated for another neuroimmune disease, Guillain-Barré syndrome (GBS). However, while the effectiveness of eculizumab for NMOSD is extremely high in many cases, there are some cases of myasthenia gravis and GBS in which eculizumab has little or no efficacy. Development of effective biomarkers that reflect complement activation in these diseases is therefore important. To identify biomarkers that could predict disease status, we retrospectively analyzed serum levels of complement factors in 21 patients with NMOSD and 25 patients with GBS. Ba, an activation marker of the alternative complement pathway, was elevated in the acute phases of both NMOSD and GBS. Meanwhile, sC5b-9, an activation marker generated by the terminal complement pathway, was elevated in NMOSD but not in GBS. Complement factor H (CFH), a complement regulatory factor, was decreased in the acute phase as well as in the remission phase of NMOSD, but not in any phases of GBS. Together, these findings suggest that complement biomarkers, such as Ba, sC5b-9 and CFH in peripheral blood, have potential utility in understanding the pathological status of NMOSD.

## Introduction

The complement system plays important roles in the innate immune system, which protects the body from foreign pathogens (1). However, when the regulatory mechanisms of complement activation are disrupted, dysregulated complement activation damages autologous cells and causes diseases such as paroxysmal nocturnal hemoglobinuria (PNH) and atypical hemolytic uremic syndrome (aHUS) (2, 3). Eculizumab, a humanized anti-C5 monoclonal antibody, is effective against PNH and aHUS (3). It specifically inhibits production of anaphylatoxin C5a and subsequent formation of the membrane attack complex (MAC), suppressing pathological complement activation. Its effectivity has been shown against neuroimmune diseases such as myasthenia gravis (MG) and neuromyelitis optica spectrum disorders (NMOSD) (4).

In MG (5) and NMOSD (6, 7), autoantibodies against acetylcholine receptor and aquaporin-4 (AQP4), respectively, activate the complement system, causing neurological symptoms due to destruction of the nervous system by the terminal complement pathway. Eculizumab is effective against these diseases and has been clinically applied (8, 9). Guillain-Barré syndrome (GBS) is also a neuroimmune disease, in which anti-ganglioside autoantibodies are produced after infection with *Campylobacter jejuni* or other organisms, and damage to the myelin sheath causes peripheral neuropathy (10). Clinical application of eculizumab for GBS is currently under investigation (11). Although eculizumab is effective in MG and GBS, some cases are non-responders, and the basis for non-response is unknown (4).

In these diseases, autoantibody titers do not correlate with disease pathology, and accurate biomarkers for complement activation could be useful not only in determining disease severity, but also in determining the potential utility of anti-complement drugs. However, biomarkers that accurately reflect complement activation in the pathogenesis of neurological diseases have not yet been identified. NMOSD and GBS are characterized by activation of the classical complement pathway. In the present retrospective cohort study, however, we measured serum levels of complement-activated markers and complement regulators involved in the alternative or terminal complement pathway in NMOSD and GBS for three reasons. First, eculizumab, which blocks the C5 cleavage involved in the initiation of the terminal complement pathway, is effective in these diseases, so activation of the alternative complement pathway and the formation of MAC in the terminal complement pathway would be expected to cause development of these diseases. Second, although the autoantibodies in NMOSD constantly exist in blood and may always activate the classical complement pathway, symptoms of NMOSD appear suddenly and recurrently, suggesting that the appearance of symptoms requires further complement activation by the alternative complement pathway in addition to the classical complement pathway. Third, in transplant-associated thrombotic microangiopathy (TA-TMA), which is thought to be a disease involving the classical and lectin complement pathways, our group previously demonstrated that abnormally high levels of plasma complement factor Ba fragment (Ba), a biomarker of activation of the alternative pathway, can be used to predict TA-TMA development and non-relapse mortality (12). We examined whether biomarkers that predict activation of the alternative and terminal complement pathways could therefore also be associated with disease pathogenesis, prognosis, and status.

## Methods

### Patients and healthy controls

Patients with NMOSD and GBS treated at Wakayama Medical University Hospital or Kindai University Hospital between 2016 and 2021 were included, and cases with both acute- and remission-paired sera archived were retrospectively selected and enrolled. Medical information was collected from medical charts. Diagnostic criteria were the 2015 international diagnostic criteria for NMOSD (13) and the Asberry diagnostic criteria for GBS (14). Seventy healthy Japanese adults, consisting of 35 males (age, mean ± SD: 45.7± 10.3 years; range: 26-68 years) and 35 females (age, mean ± SD: 44.7± 12.3 years; range: 27-75 years) were enrolled as healthy controls (15).

### Definitions of acute and remission phases of NMOSD

NMOSD relapse was defined based on criteria from previous clinical studies (8). Briefly, new onset or worsening neurologic symptoms must persist >24 hours and should not be attributable to confounding clinical factors. Remission was defined as a period when neurologic symptoms were stable for at least one month, and no new lesions shown on MRI imaging.

### Evaluation of acute and remission phases of GBS

The acute phase of GBS was defined as the peak of symptoms prior to treatment. The stable phase was defined as a time when symptoms became mild and stable following treatment. Disabilities were evaluated using the Hughes functional grade scale (11).

### Measurement of anti-AQP4 and anti-ganglioside antibodies

Anti-AQP4 antibodies titers were analyzed using a cell-based assay with live human embryonic kidney 293 cells stably transfected with the M23 isoform of AQP4. Goat anti-human IgG Fc labelled with DyLight 488 (Thermo Fisher Scientific, Waltham, MA) was used as a secondary antibody after the transfected cells were exposed to the patients’ diluted sera. Anti-ganglioside antibodies were examined by ELISA. Serum IgG antibodies to 11 glycolipid antigens (GM1, GM2, GM3, GD1a, GD1b, GD3, GT1b, GQ1b, GT1a, Gal-C, and GalNAc-GD1a) were analyzed.

### Complement measurement

Serum samples obtained from patients and healthy controls were stocked until analysis at -80°C. Serum levels of sC5b-9 and Ba were measured using MicroVue SC5b-9 Plus EIA and MicroVue Ba EIA, respectively (Quidel, San Diego, CA). Serum levels of complement factor H (CFH) and complement factor I (CFI) were measured using ELISA kits (Abnova, Taipei, Taiwan). Complement data from 70 healthy Japanese volunteers (age: 26–75 years) were used as healthy controls, and reference ranges of complement markers (average levels ± 2 S.D.) in their serum were defined as previously described (15). The normal ranges of serum for sC5b-9 and Ba have been found to be greater than that of EDTA plasma, but the ranges in serum stored at -80 °C until analysis confirmed stability, even after five freeze-thaw cycles. In the present study, we compared the patients data with previous data of 70 healthy Japanese adults as controls.

### Statistical analysis

Statistically significant differences were evaluated between three groups (healthy controls, patients with NMOSD and patients with GBS) using a one-way analysis of variance (ANOVA) and a Tukey-Kramer test as a *post hoc* test, and between two groups (the acute and remission phases) using a paired t-test. *P* < 0.05 (two-tailed) was considered significant for all results. Pearson correlation analysis was performed using JMP pro 16.0 software.

## Results

We retrospectively analyzed 21 patients with NMOSD (19 females and 2 males) and 25 patients with GBS (14 females and 11 males) (**Table 1**). The mean age at the time of blood collection in the acute phase of NMOSD was 48.0 years, mean duration of illness was 5.1 years, and mean expanded disability status scale (EDSS) was 5.3. Mean EDSS during NMOSD remission was 4.5. The mean age at GBS onset was 50.8 years, and the mean severity of illness was Hughes functional grade scale 3.4. Mean Hughes functional grade scale during the remission phase of GBS (at discharge) was 1.7. Anti-AQP4 and anti-glycolipid autoantibodies were positive in 81% patients with NMOSD and 88% patients with GBS, respectively.

**Table 1:** Patient backgrounds.

		NMOSD (n = 21)	GBS (n = 25)	*p*-values
Sex (female/male)		19/2	14/11	< 0.01
Age, mean ± SD [range] (y)		48.0 ± 2.5 [17–74]	50.8 ± 4.4 [14–77]	NS
Disease duration, mean ± SD [range] (y)		5.1 ± 1.2 [0–14]	NA	
Anti-aquaporin 4 antibody-positive		17 (81%)	NA	
Anti-glycolipid antibody-positive		NA	22 (88%)	
Lesions according to MRI findings	Optic nerve	4 (19.0%)	NA	
	Spinal cord	17 (81.0%)	NA	
	Brain	2 (9.5%)	NA	
EDSS, Mean ± SD [range]	Acute phase	5.3 ± 2.1 [2–8.5]	NA	
	Remission	4.5 ± 2.2 [2–8.0]	NA	
Functional grade, Mean ± SD (AU)	Acute phase	NA	3.4 ± 1.0	
	Remission	NA	1.7 ± 0.8	

NMOSD, neuromyelitis optica spectrum disorders; GBS, Guillain-Barré syndrome; SD, standard deviation; EDSS, expanded disability status scale; MRI, magnetic resonance imaging; NA, not applicable; NS, not significant.

**Figure 1:**
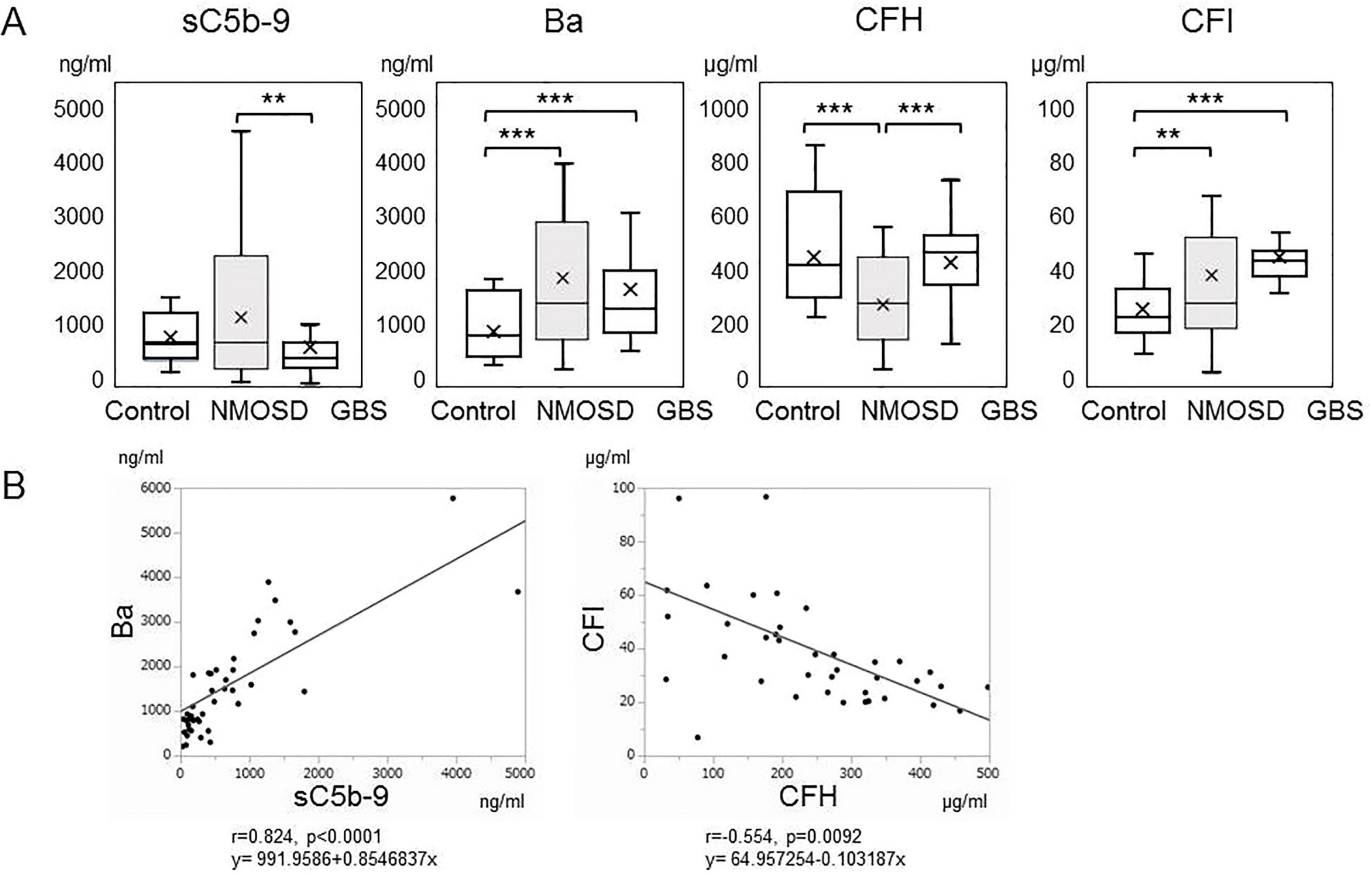
**(A)** Serum levels of complement markers in the acute phases of neuromyelitis optica spectrum disorders and Guillain-Barré syndrome. Serum levels of sC5b-9, Ba, complement factor H, and complement factor I in the acute phases of neuromyelitis optica spectrum disorders and Guillain-Barré syndrome, together with those of healthy controls, are shown by box plots. **p < 0.01, and ***p < 0.001, ANOVA and Tukey-Kramer test as a *post hoc* test. **(B)** Correlation analysis of complement markers in the acute phase of neuromyelitis optica spectrum disorders. The relevance of serum levels of sC5b-9, Ba, complement factor H and complement factor I in the acute phase of neuromyelitis optica spectrum disorders were analyzed. Ba and sC5b-9 (r=0.824, *p*<0.00010), and complement factor H and complement factor I (r=-0.554, *p*=0.0092) showed positive and negative correlations, respectively.

sC5b-9, an activation marker generated by the terminal complement pathway, was significantly higher in the acute phase of NMOSD compared with in the acute phase of GBS (**Figure 1A**). Activation of the complement system was thus indicated to have progressed to the terminal complement pathway in the acute phase of NMOSD. Serum Ba, an activation marker of the alternative complement pathway, was also higher in the acute phases of both NMOSD and GBS compared with healthy controls (**Figure 1A**).

Subsequently, we measured complement regulatory protein levels in NMOSD and GBS. CFH was within the reference range but significantly lower in patients with NMOSD than in healthy controls or in patients with GBS (**Figure 1A**). However, CFI, another complement regulatory protein, was higher in patients with NMOSD and in patients with GBS than in healthy controls.

To determine the correlations of these biomarkers with each other in NMOSD, we performed a correlation analysis (**Figure 1B**). Ba and sC5b-9 levels (r=0.824, *p*<0.00010), and CFH and CFI levels (r=-0.554, *p*=0.0092) showed positive and negative correlations, respectively. However, no other correlations were detected in samples obtained from patients in the acute phase of NMOSD.

**Figure 2:**
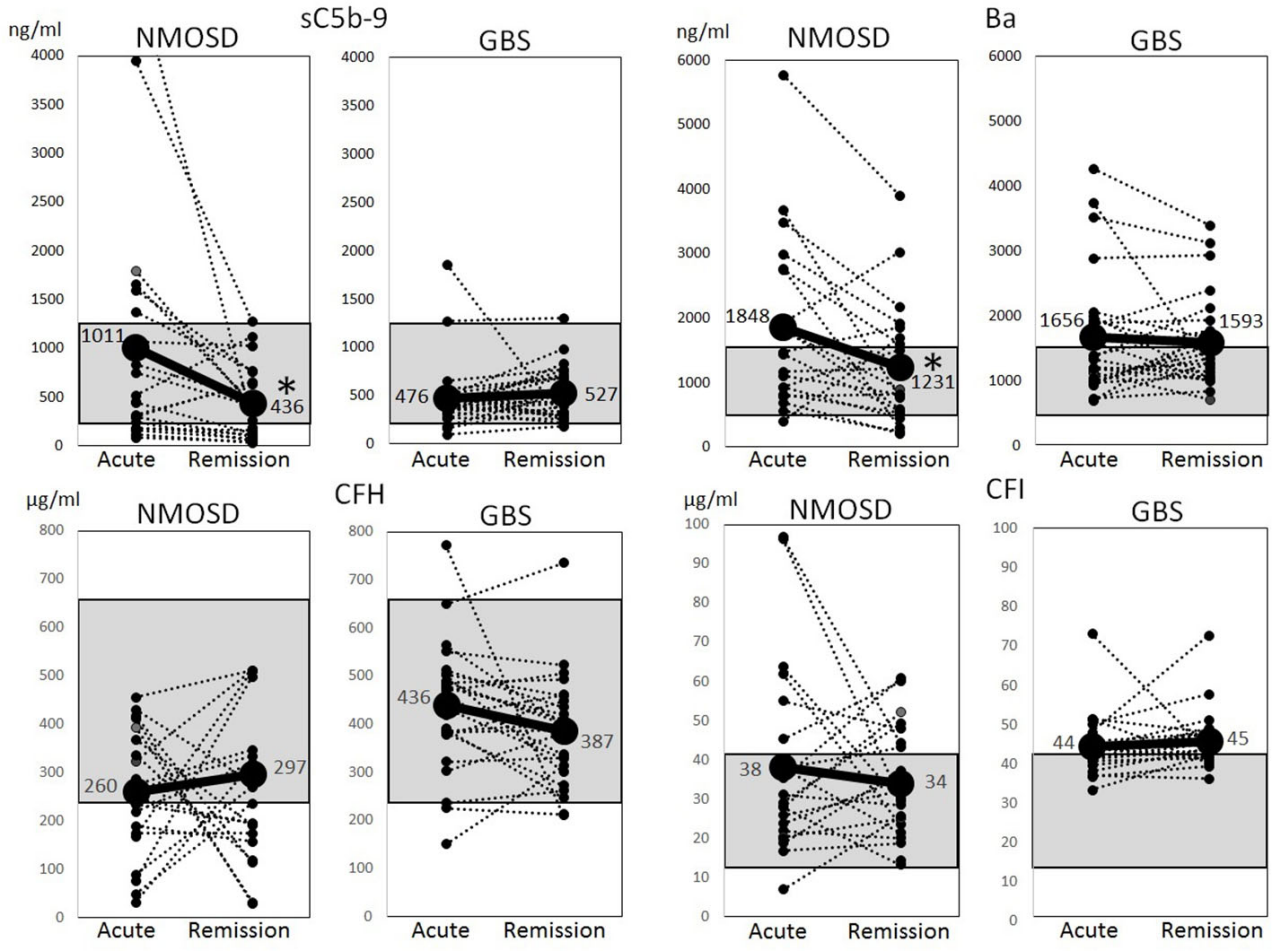
Changes of complement markers during the acute and remission phases of neuromyelitis optica spectrum disorders and Guillain-Barré syndrome. Changes of serum levels of sC5b-9, Ba, complement factor H and complement factor I during the acute and remission phases of neuromyelitis optica spectrum disorders and Guillain-Barré syndrome were analyzed. The dotted lines indicate changes in individual cases, and solid lines indicate changes in average levels. The gray shadow indicates reference ranges in healthy Japanese adults (sC5b-9: 181–1266 ng/ml, Ba: 438–1546 ng/ml, complement factor H: 238–663 µg/ml, complement factor I: 11–42 µg/ml) (15). *p < 0.01, paired t-test.

The above-mentioned complement factors examined in the acute phase were also analyzed for changes in the remission phase. The main laboratory data did not change between the acute and remission phases (**Table 2**). The sC5b-9 and Ba markers, which were elevated in the acute phase of NMOSD, decreased significantly in the remission phase (**Figure 2**). Although CFH levels were increased in the remission phase of 12 patients with NMOSD, the average levels of CFH still remained lower than the healthy control level during the remission phase as well as during the acute phase. Moreover, in some patients, CFH levels were markedly reduced in the remission phase. The levels of CFI were decreased in 10 patients in the remission phase of NMOSD, but the average levels of CFI were still higher than those of healthy controls during the remission phase. To rule out these changes of complement markers being due to previously-received treatments, we analyzed complement markers in 10 patients that had not received any treatment at the time of the first-episode of NMOSD and obtained similar results (**Supplementary Table 1**, **Supplementary Figures 1, 2**). However, in patients with GBS, sC5b-9, Ba, CFH, and CFI did not change between the acute and remission phases, and Ba and CFI in the remission phase remained higher than those in the healthy controls (**Figure 2**).

**Table 2:** Laboratory data and treatments of the patients with NMOSD.

	Acute phase	Remission	*p*-values
Blood Tests			
White Blood Cells (/μL)	7223 ± 2187	7499 ± 3132	0.912
Neutrophils (/μL)	5138 ± 2169	4803 ± 2735	0.849
Lymphocytes (/μL)	1420 ± 639	2019 ± 1527	0.364
Monocytes (/μL)	544 ± 354	548 ± 253	0.983
Albumin (g/dL)	4.2 ± 0.4	3.8 ± 0.6	0.807
CRP (mg/dL)	0.367 ± 0.666	0.199 ± 0.485	0.410
Cerebrospinal fluid test			
Cell count (/mm^3^)	13.7 ± 28.7	3.0 ± 3.0	0.621
Protein (mg/dL)	46.8 ± 37.4	36.6 ± 20.7	0.144
Treatments at blood collection			
None	10 (47.6%)	8 (38.1%)	
Steroids	6 (28.6%)	6 (28.6%)	
Immunosuppressants	1 (4.8%)	2 (9.5%)	
Steroids + Immunosuppressants	3 (14.3%)	4 (19.0%)	
Plasma exchange	1 (4.8%)	1 (4.8%)	

Data are shown as mean ± standard deviation. CRP, C-reactive protein; NMOSD, neuromyelitis optica spectrum disorders.

We detected no correlations between levels of complement markers and most of the clinical manifestations, disease severity, or cerebrospinal fluid test values in the acute phase of NMOSD. There was, however, a moderate positive correlation between levels of CFI and disease duration (r=0.520) (**Supplementary Table 2**).

## Discussion

In the present study, we measured serum levels of Ba, sC5b-9, CFH, and CFI in the acute and remission phases of NMOSD and GBS. In NMOSD, we identified that sC5b-9 and Ba levels correlated significantly with clinical stage, suggesting that activation of the alternative and terminal complement pathways contributes to exacerbation of NMOSD. The levels of sC5b-9 and Ba may be influenced by the types of treatments and whether they were obtained at the time of the first-episode or after some treatments, but similar results were also obtained in the 10 patients who had not received any treatment at the time of the first-episode. Furthermore, the increased levels of Ba and sC5b-9 were strongly correlated, suggesting that activation of the classical complement pathway by autoantibodies in the periphery led to activation of the alternative and terminal complement pathway. In addition to increased levels of C5a in cerebrospinal fluid that were previously reported as a biomarker of NMOSD (16), the present findings suggest that sC5b-9 and Ba levels in peripheral blood could be useful markers in determining whether NMOSD is in the active stage. NMOSD is known to be caused by injury to astrocytes which express AQP4 (17). Circulating anti-AQP4 antibodies must destroy the brain-blood barrier (BBB) in order to reach astrocytes. IL-6 (18), anti-glucose-regulated protein 78 autoantibodies (19), and polymorphonuclear leukocytes (20) have been reported to be involved in the disruption of BBB. Complement activation in the periphery may also contribute to the destruction. The involvement of peripheral complement activation in the pathogenesis of NMOSD using animal models should be clarified in future studies.

CFH was decreased during both acute and remission phases of NMOSD. There are three possible reasons for these decreased levels. First, AQP4 is expressed not only in astrocytes, but also in muscle and renal tubules, and anti-AQP4 antibodies react with them to activate complement in the periphery. CFH may therefore be consumed and reduced in NMOSD to control activation of the complement system. In the present study, CFH levels in 12 patients were increased in the remission phase. A second possible reason for the decreased levels is that NMOSD could be originally caused in individuals with low CFH levels and activation of alternative and terminal complement pathways initiated by anti-AQP4 autoantibodies might not be adequately suppressed by low CFH levels. Eculizumab, which blocks the C5 cleavage involved in the initiation of the terminal complement pathway, is an effective treatment for almost all NMOSD cases with anti-AQP4 autoantibodies (8). In patients with NMOSD, low CFH levels may be a significant cause of complement activation in the periphery. A third possible reason for the decreased levels could be that CFH production may be suppressed by steroid or immunosuppressive therapies. In some patients, remarkably decreased levels of CFH were observed in the remission phase.

In NMOSD, modest increase of CFI levels was also observed, and the levels of CFH and CFI had negative correlation.We detected a moderate positive correlation between CFI levels and disease duration, so CFI may increase by inflammation induced in the acute phase to block activation of the complement system in the periphery.

In GBS, there were no significant differences in sC5b-9, Ba, CFH, or CFI levels between the acute and remission phases. In addition, in the acute phase of GBS, Ba was increased but sC5b-9 was unchanged, suggesting that activation of classical complement pathway by autoantibodies led to activation of the alternative pathway in the periphery, but did not progress to the terminal complement pathway. The levels of CFH and CFI remained high in both acute and remission phases of GBS, suggesting that their regulatory functions would be maintained. Therapies targeting complement pathways other than the terminal complement pathway could therefore be effective in cases of GBS without elevated sC5b-9 levels. Alternatively, anti-C5 antibodies could be effective in cases of GBS with elevated sC5b-9.

Comprehensive measurement of complement biomarkers such as Ba, sC5b-9, and CFH could contribute to delineating the pathogenesis and pathological status of NMOSD. The complement biomarkers in cerebrospinal fluid should also be measured to clarify the contribution to the pathogenesis of NMSD. We will also analyze the complement biomarkers in patients treated with eculizumab in a future study to determine whether these could be predictive biomarkers for response to eculizumab treatment. The present study was performed retrospectively using previously collected serum samples, so the reference ranges were too broad to determine valid cut-off values of Ba and sC5b-9 for prediction of acute and remission phases. However, this study suggests that the results should be validated in a future prospective study using plasma treated with ethylenediaminetetraacetic acid-disodium salt.

## Data availability statement

The raw data supporting the conclusions of this article will be made available by the authors, without undue reservation.

## Ethics statement

The studies involving human participants were reviewed and approved by The study was approved by Research Ethics Committee of Wakayama Medical University (approval number: G154 and 3278) and Kindai University (approval number: 2021-164). Written informed consent for participation was not required for this study in accordance with the national legislation and the institutional requirements.

## Author contributions

KM, NI, and HI contributed to the conception and design of the study. KM, MM, KO, NW, and MK contributed to acquisition and analysis of data. KM, KK and NI contributed to drafting of the text and preparation of the figures. HT contributed to statistical analyses of data. All authors contributed to the article and approved the submitted version.
