# Supplementary material for: Complement biomarkers reflect the pathological status of neuromyelitis optica spectrum disorders
Source: Front Immunol. 2023 Mar 3;14:1090548. doi: 10.3389/fimmu.2023.1090548 (PMC10020620; doi:10.3389/fimmu.2023.1090548)
Supplement: Supplementary file 1 [file DataSheet_1.pdf]

Supplementary Material

Supplementary Figure 1.

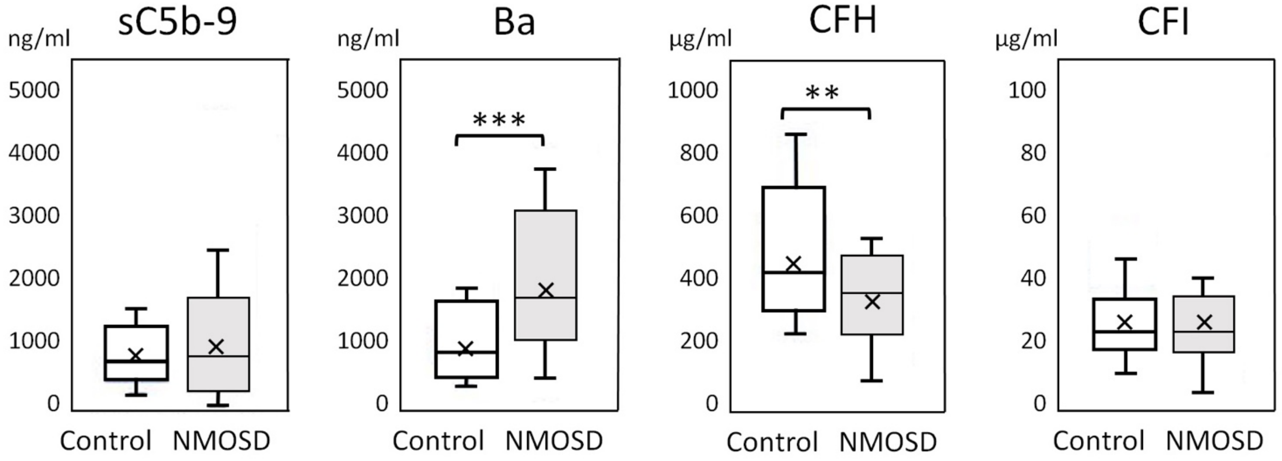

Serum levels of complement markers of the acute phase in 10 naïve patients with neuromyelitis optica spectrum disorders

Serum levels of sC5b-9, Ba, complement factor H (CFH), and complement factor I (CFI) in the acute phases of 10 patients that had not received any treatment at the time of the first-episode of neuromyelitis optica spectrum disorders, together with those of healthy controls, are shown by box plots.

**p < 0.01, and ***p < 0.001, Student t-test.

Supplementary Figure 2.

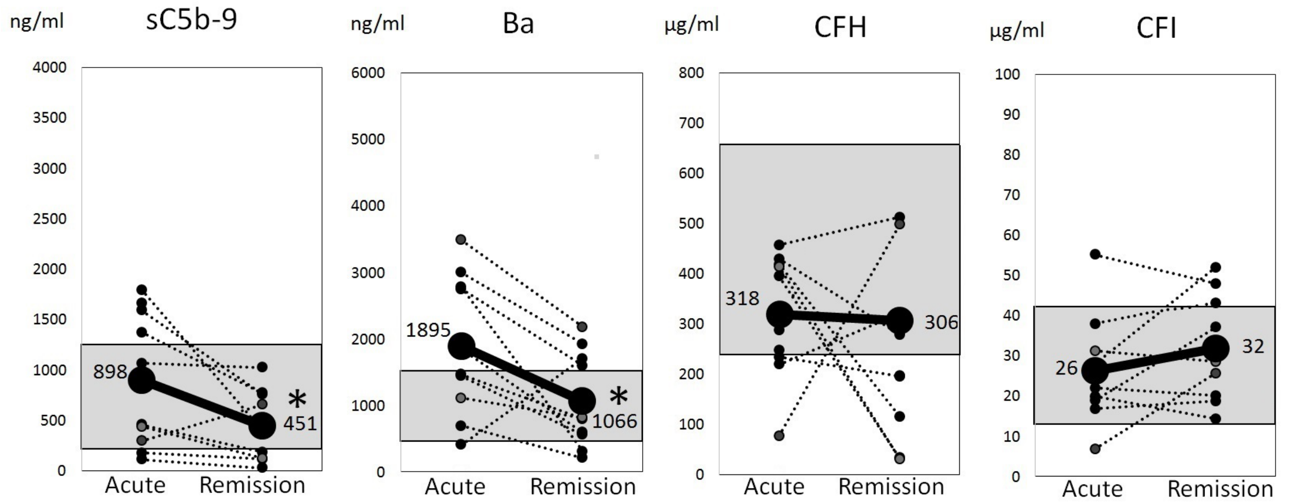

Changes of complement markers during the acute and remission phases of 10 naïve patients with neuromyelitis optica spectrum disorders

Changes of serum levels of sC5b-9, Ba, complement factor H (CFH) and complement factor I (CFI) during the acute and remission phases of 10 patients that had not received any treatment at the time of the first-episode of neuromyelitis optica spectrum disorders were analyzed. The dotted lines indicate changes in individual cases, and solid lines indicate changes in average levels. The gray shadow indicates reference ranges in Japanese healthy adults (sC5b-9: 181–1266 ng/ml, Ba: 438–1546 ng/ml, CFH: 238–663 μg/ml, CFI: 11–42 μg/ml) (15). **p* < 0.01, paired t-test.

Supplementary Table 1. Laboratory Data and Treatments in NMOSD-naïve patients

	Acute phase	Remission	*p*-values
Blood Tests			
White Blood Cells (/μL)	7535 ± 2320	7492 ± 3819	0.991
Neutrophils (/μL)	5203 ± 2089	4224 ± 1582	0.698
Lymphocytes (/μL)	1582 ± 729	2561 ± 1976	0.420
Monocytes (/μL)	580 ± 471	529 ± 238	0.873
Albumin (g/dL)	4.2 ± 0.4	3.8 ± 0.4	0.865
CRP (mg/dL)	0.600 ± 0.861	0.339 ± 0.685	0.538
Cerebrospinal fluid test			
Cell count (/mm^3)	22.9 ± 40.4	2.9 ± 3.7	0.246
Protein (mg/dL)	40.9 ± 14.4	31.4 ± 5.6	0.654
Treatments at blood collection			
None	10 (100%)	5 (50.0%)	
Steroids	0 (0.0%)	3 (30.0%)	
Immunosuppressants	0 (0.0%)	1 (10.0%)	
Steroids + Immunosuppressants	0 (0.0%)	1 (10.0%)	
Plasma exchange	0 (0.0%)	0 (0.0%)	

Data are shown as mean ± standard deviation. Abbreviations: CRP = C-reactive protein; NMOSD = neuromyelitis optica spectrum disorders.

Supplementary Table 2. Correlations between serum complement marker levels and clinical data in the acute phase of NMOSD

	sC5b-9	Ba	CFH	CFI
Age	-0.175	-0.167	0.036	-0.008
Disease duration	0.085	-0.026	-0.316	<u>0.520</u>
EDSS	0.140	-0.032	-0.248	0.183
White Blood Cells	0.125	0.004	0.042	0.176
Neutrophils	0.033	0.019	-0.070	0.189
Lymphocytes	0.033	-0.096	0.259	-0.013
Monocytes	0.041	-0.024	0.177	0.014
Albumin	0.154	0.059	-0.198	0.128
CRP	-0.040	-0.001	0.399	-0.396
CSF cell count	0.104	0.180	-0.074	-0.179
CSF protein	-0.228	-0.249	-0.148	0.481

Data are shown as r value. Abbreviations: CSF=cerebrospinal fluid; NMOSD=neuromyelitis optica spectrum disorders; EDSS = expanded disability status scale. Underline: significant correlation
